# Combined effects of melatonin and FGF-2 on mouse preosteoblast behavior within interconnected porous hydroxyapatite ceramics - *in vitro* analysis

**DOI:** 10.1590/1678-775720150606

**Published:** 2016

**Authors:** Mohammad Zeshaan RAHMAN, Hideo SHIGEISHI, Kazuki SASAKI, Akira OTA, Kouji OHTA, Masaaki TAKECHI

**Affiliations:** Hiroshima University, Institute of Biomedical and Health Sciences, Department of Oral and Maxillofacial Surgery, Hiroshima, Japan.

**Keywords:** Hydroxyapatite, Fibroblast growth factor-2, Melatonin

## Abstract

**Objective:**

Biocompatible materials such as interconnected porous hydroxyapatite ceramics (IP-CHA) loaded with osteogenic cells and bioactive agents are part of an evolving concept for overcoming craniofacial defects by use of artificial bone tissue regeneration. Amongst the bioactive agents, melatonin (MEL) and basic fibroblast growth factor (FGF-2) have been independently reported to induce osteoblastic activity. The present *in vitro* study was undertaken to examine the relationship between these two bioactive agents and their combinatory effects on osteoblastic activity and mineralization *in vitro*.

**Material and Methods:**

Mouse preosteoblast cells (MC3T3-E1) were seeded and cultured within cylindrical type of IP-CHA block (ø 4x7 mm) by vacuum-assisted method. The IP-CHA/MC3T3 composites were subjected to FGF-2 and/or MEL. The proliferation assay, alkaline phosphatase enzyme activity (ALP), mRNA expressions of late bone markers, namely Osteocalcin (OCN) and Osteopontin (OPN), and Alizarin Red staining were examined over a period of 7 days.

**Results:**

FGF-2 mainly enhanced the proliferation of MC3T3-E1 cells within the IP-CHA constructs. MEL mainly induced the mRNA expression of late bone markers (OCN and OPN) and showed increased ALP activity of MC3T3 cells cultured within IP-CHA construct. Moreover, the combination of FGF-2 and MEL showed increased osteogenic activity within the IP-CHA construct in terms of cell proliferation, upregulated expressions of OCN and OPN, increased ALP activity and mineralization with Alizarin Red. The synergy of the proliferative potential of FGF-2 and the differentiation potential of MEL showed increased osteogenic activity in MC3T3-E1 cells cultured within IP-CHA constructs.

**Conclusion:**

These findings indicate that the combination of FGF-2 and MEL may be utilized with biocompatible materials to attain augmented osteogenic activity and mineralization.

## INTRODUCTION

Bone grafting plays an essential role in craniofacial surgery performed for both reconstructive and aesthetic purposes, which has led to discovery of different biomaterials, including hydroxyapatite (HAp), a member of the non-resorbable calcium phosphate group of biomaterials^23^. HAp has been formed into a variety of shapes and dimensions, and shown both biocompatibility and osteoconductivity since the discovery of its similarities with natural bone^23^. Porous type HAp ceramics are expected to facilitate bone formation and become integrated with host bone tissue. However, the pores of HAp are not fully replaced with new bone for a substantial period of time due to limited interconnection, which eventually leads to undesirable results^1^. The interconnection of pores is considered to be an essential factor for osteoconduction inside an HAp ceramic appliance for cell viability and function. To overcome this problem, interconnected porous HAp ceramics (IP-CHA) components with fully interconnected and symmetrical pores have been developed^21^. This unique structure provides extensive incorporation with host cells more rapidly than other types of porous calcium HAp ceramic, while its porous architecture also provides optimum compressive strength of up to 12 MPa, similar to cancellous bone^21^. It has been suggested that IP-CHA may have an additional osteoinductive advantage if the porous architecture could be utilized to transplant osteoinductive agents or osteogenic cells or both.

Fibroblast growth factor 2 (FGF-2) is a versatile member of the 23-polypeptide growth factor family^13^. It has been found to participate in a variety of biological processes, such as angiogenesis, hematopoiesis, cell growth and bone development^7^ ^,^ ^13^ ^,^ ^16^. FGF-2 has been reported to stimulate osteoblast proliferation rather than differentiation in immature cells^19^. In addition, FGF-2-induced cell signaling enhance the proliferation and growth of osteoblastic MC3T3-E1 cells^5^, suggesting that FGF-2 plays an important role in cell growth of osteoblasts.

Melatonin (MEL) is a pineal hormone that is also synthesized from other human cells and organs, such as the retina, bone marrow, and gastrointestinal tract^2^ ^,^ ^10^ ^,^ ^18^. Roth, et al.^17^ (1999) demonstrated the direct effects of MEL on differentiation of rat preosteoblast cells, while it has also been reported to inhibit RANKL-induced bone resorption and thereby promote bone formation^14^. These observations imply that MEL positively influences bone formation.

Taken together, FGF-2 is involved in the proliferation of osteoblasts and MEL is thought to promote bone formation. The combination of FGF-2 and MEL may more effectively induce increased activity of osteoblast. We have previously reported that FGF-2 and MEL play a significant role in osseointegration around titanium implants in animal models^20^. However, their combined effects on osteoblast cell growth and bone formation have not been fully elucidated *in vitro*. Also, their combination within useful biomaterials, such as HAp, remains undocumented. Hence, we investigated the effects of FGF-2 and MEL in combination on mouse preosteoblast cells when cultured within an IP-CHA construct.

## MATERIAL AND METHODS

### Cell culture & IP-CHA

MC3T3-E1 mouse preosteoblast cells were cultured in α-minimal essential medium (α-MEM) (Sigma-Aldrich, St. Louis, MO, USA) supplemented with 10% fetal bovine serum (FBS) (Biowest, Miami, FL, USA), 1% penicillin-streptomycin, and L-Glutamate, and incubated with 5% CO_2_ at 37**°**C.

We used a cylindrical type of porous IP-CHA block (NEOBONE^®^, MMT, Osaka, Japan) that was 4 mm in diameter, 7 mm in height, with 75% porosity. The mean pore diameter was 150 µm and the pores interconnections were 40 µm. Prior to cell seeding, IP-CHA blocks were pre-coated with cell-free medium to enhance cell adhesion in the interior of the scaffold. Medium was trickled onto the block and then it was subjected to vacuum, which moved the air out of the porous IP-CHA and drew medium in.

Once the cells were sub-confluent, viability was determined by trypan blue staining. Next, 1x10^5^ viable cells were resuspended in 130 µl of expansion medium and concentrated cell suspensions were pipetted onto the IP-CHA in a 24-well plate. To ensure cell penetration within the IP-CHA construct, each one was subjected to vacuum of 100 mmHg for 100 milliseconds^4^. The samples were then placed in an incubator for 1 hour to allow the cells to adhere to the interior of the construct. An additional 1.5 ml of expansion medium was added later to the IP-CHA/MC3T3 composite to aid proliferation within the scaffold for 24 hours before treating with FGF-2 and/or MEL.

### Scanning electron microscope

Cells on the scaffolds were fixed with 2.0% glutaraldehyde in PBS for 30 minutes. After washing the scaffolds with PBS and distilled water, these were subjected to dehydration with ethanol series (50%, 60%, 70%, 85%, 90%, 95% and 100%). The presence of MC3T3-E1 cells 3.5 mm deep by horizontal section inside IP-CHA was examined by scanning electron microscope (SEM) (VE-8800, Keyence, Osaka, Japan) at 15 kV of accelerating voltage after a gold coating.

### FGF-2 and MEL treatment

We determined the optimum concentration of FGF-2 and MEL based on the data from the previous reports^15^ ^,^ ^24^. Mouse recombinant FGF-2 (Sigma-Aldrich) was used to evaluate the optimum concentration of FGF-2 needed for cell proliferation, therefore the samples were subjected to different concentrations of FGF-2 (2, 20, 100 µg/ml)^24^. MEL (Sigma-Aldrich) was used to evaluate the optimum concentration of MEL needed for cell differentiation, therefore the samples were subjected to different concentrations of MEL (50, 200, 1000 nM)^15^.

### Proliferation assay

Cell proliferation with FGF-2 and/or MEL treatment was evaluated using an MTS Assay (Aqueous One Cell Proliferation Assay, Promega, Madison, WI, USA) after 1, 3, and 5 days. The principle behind the MTS assay is the formation of formazan crystals by dehydrogenase enzyme in functionally active cell mitochondria. The amount of purple formazan formed is directly proportional to the number of viable cells. The method was performed according to the manufacturer’s protocol. Briefly, the IP-CHA/MC3T3 composite was treated with and without FGF-2 and/or MEL, then gently rinsed in PBS and transferred to a new 24-well plate. MTS solution (100 µl *per* 1 ml of expansion medium) was added to the composite and subjected to vacuum at 100 mmHg for 100 milliseconds to ensure that the MTS solution entered the core. Next, the composite was allowed to incubate for 2 hours, after which medium in the wells was gently aspirated and discarded. Finally, 750 µl of dimethyl sulfoxide was added for dissolving the formazan crystals formed by the cells in the composite and 250 ml of this solution was transferred to a 96-well plate, and absorbance at 490 nm was measured using a microplate reader (Bio-Rad, Hercules, CA, USA). Results are expressed as the mean±SD of 3 independent experiments.

### Quantitative RT-PCR analysis

RNA was extracted using an RNAeasy micro kit (Qiagen, Hilden, Germany). Reverse transcription into cDNA was performed using SuperScript III First Strand Synthesis Supermix (Invitrogen, Carlsbad, CA, USA). Quantification of mRNA levels was carried out using Eppendorf Master Cycler and SYBR Green Master Mix (TOYOBO, Tokyo, Japan). The reaction mixture consisted of 1.0 µg of cDNA, 12 µl of SYBR Green Mix, and 10 µmol of each pair of oligonucleotide primers. GAPDH was used as the reference mRNA control. The PCR protocol was as follows: initial melting at 95°C for 10 minutes, followed by 40 cycles at 95°C for 15 seconds, 60°C for 30 seconds, and 72°C for 40 seconds. Reverse transcribed Human Total Reference RNA (Stratagene, Cheshire, UK) was used to plot a standard curve. Results are expressed as the mean±SD of 3 independent experiments.

### ALP enzyme activity

ALP enzyme activity was determined using an ALP assay measurement kit (TRACP & ALP Assay Kit, Takara, Osaka, Japan). Briefly, the IP-CHA/MC3T3 composites after 3, 5, and 7 days of culture with FGF-2 and/or MEL were washed 3 times in PBS, then homogenized in the provided extraction solution and sonicated for 3 minutes. Cell lysates were then collected by centrifugation at 16,000 g for 5 minutes and 50 μl of the supernatant was mixed with 50 μl of the substrate solution (p-nitro-phenyl phosphate) provided in the assay kit. The solution was then incubated at 37°C for 1 hour before measuring absorbance at 405 nm (Bio-Rad). Next, we calculated the ratio of absorbance of each sample in relation to the control sample at day 3. Results are expressed as the mean±SD of 3 independent experiments.

### Alizarin Red staining and quantification

Extracellular calcium deposits were examined by Alizarin Red staining. Briefly, Alizarin Red solution was freshly prepared by dissolving 2 g of Alizarin Red (Sigma-Aldrich) in 100 ml of deionized distilled water, then pH was incrementally adjusted to 4.1-4.3 using 0.1% NH_4_OH solution. Both monolayer cultures and treated IP-CHA/MC3T3 composites were gently washed with PBS. The cells were fixed in enough 10% neutral buffered formalin (Sigma-Aldrich) to submerge the cells or composite. After 30 minutes, formalin was gently aspirated and the cells were washed with deionized distilled water. Finally, prepared Alizarin Red solution was added to cover the cells and incubated at room temperature in the dark for 45 minutes, after which the monolayer cells were examined under a microscope. Later, both the monolayers and treated IP-CHA/MC3T3 composites were quantified. Briefly, the monolayers and treated IP-CHA/MC3T3 composites were submerged in 20% methanol and 10% acetic acid solution in water. After substantial vortexing, readings were obtained using a spectrophotometer at 450 nm of absorbance. Results are expressed as the mean±SD of 3 independent experiments.

### Statistical methods

Data obtained were analyzed using one-way analysis of variance (ANOVA) and the results are presented as the mean±standard deviation. At least 3 independent IP-CHA blocks were used for each experiment in statistical analysis. Results showing statistical significance at p<0.01 and p<0.05 are denoted by * and **, respectively, in the figures.

## RESULTS

### Optimum concentration of FGF-2 and MEL

**Figure 1:**
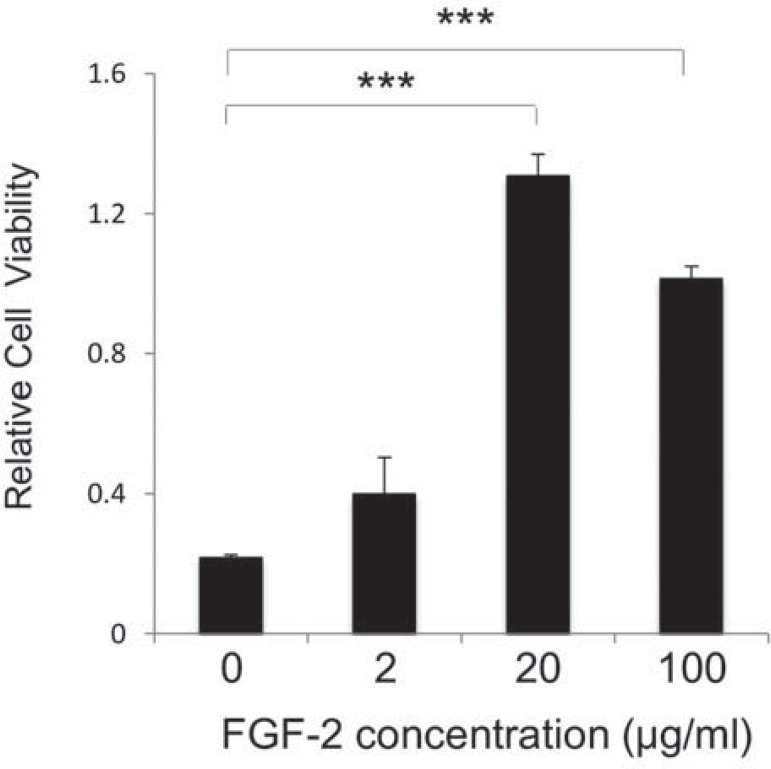
Proliferation assay of MC3T3-E1 cells at different concentrations of FGF-2. Cell growth of MC3T3-E1 cells was examined on monolayer culture using MTT assay at different concentrations of FGF-2 (0, 2, 20,100 µg/ml). The high proliferative potential of MC3T3-E1 cells was observed in the presence of 20 µg/ml of FGF-2 at day 5. Statistical significance of p<0.01 is indicated by ***

To determine the optimum concentration of FGF-2 and MEL for our study, MC3T3-E1 cells were cultured and treated accordingly. Firstly, we determined the optimum concentration of FGF-2 for the growth of MC3T3-E1 cells by MTT assay. The high proliferative potential of MC3T3-E1 cells on monolayer culture was observed in the presence of 20 µg/ml FGF-2 at day 5. Thus, we determined 20 µg/ml FGF-2 as optimum concentration in this study (Figure 1).

**Figure 2:**
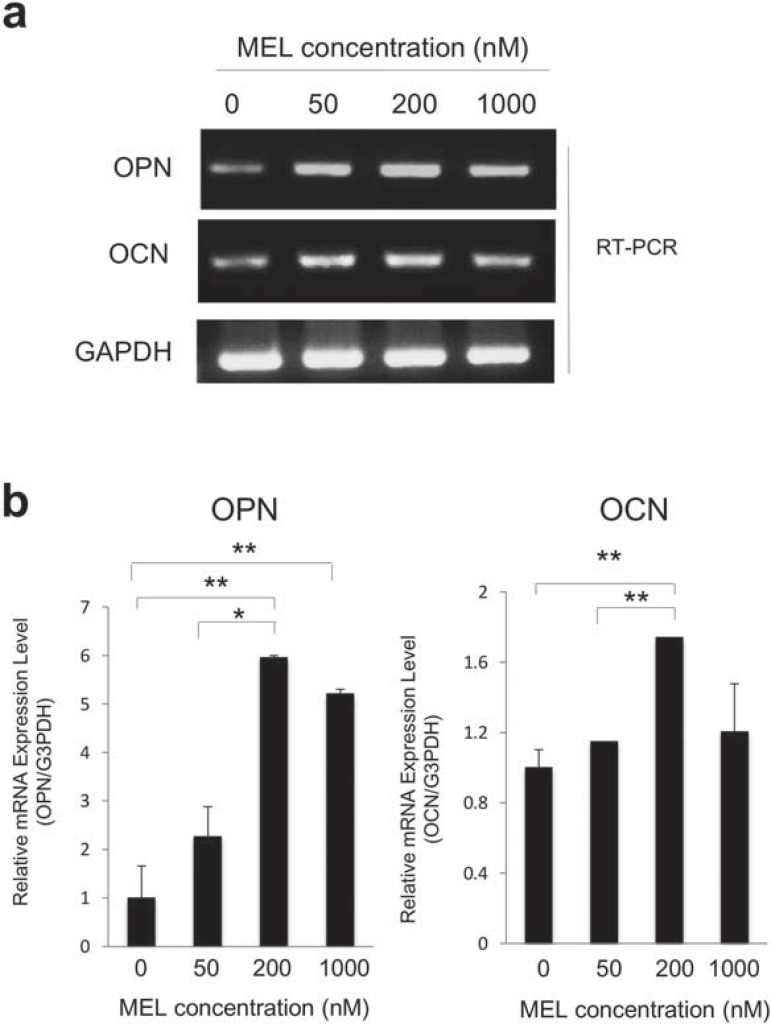
mRNA expressions of osteopontin (OPN) and osteocalcin (OCN) of MC3T3-E1 cells at different concentrations of melatonin (MEL). a- mRNA expressions of OPN and OCN were examined at different concentrations of MEL (50, 200, 1000 nM); b- Increased mRNA expression of OPN and OCN was found in the presence of 200 nM MEL. Statistical significances shown by p<0.05 and p<0.01 are indicated by * and **, respectively

Secondly, we determined the optimum concentration of MEL on monolayer cultures. Osteopontin (OPN) and osteocalcin (OCN) are considered to be late osteogenic markers, and have roles in the onset of the mineralization phase of osteoblast lineage^3^. Therefore, we examined OPN and OCN mRNA expression to determine the optimum concentration of MEL. MEL significantly induced OPN (Figure 2a) and OCN mRNA (Figure 2b) at 200 nM compared to the controls and 50 nM treatments. Therefore, 200 nM could be considered the lowest concentration of MEL that can elicit an osteoblastic response for our study.

### SEM analysis of IP-CHA/MC3T3-E1 cells composites

**Figure 3:**
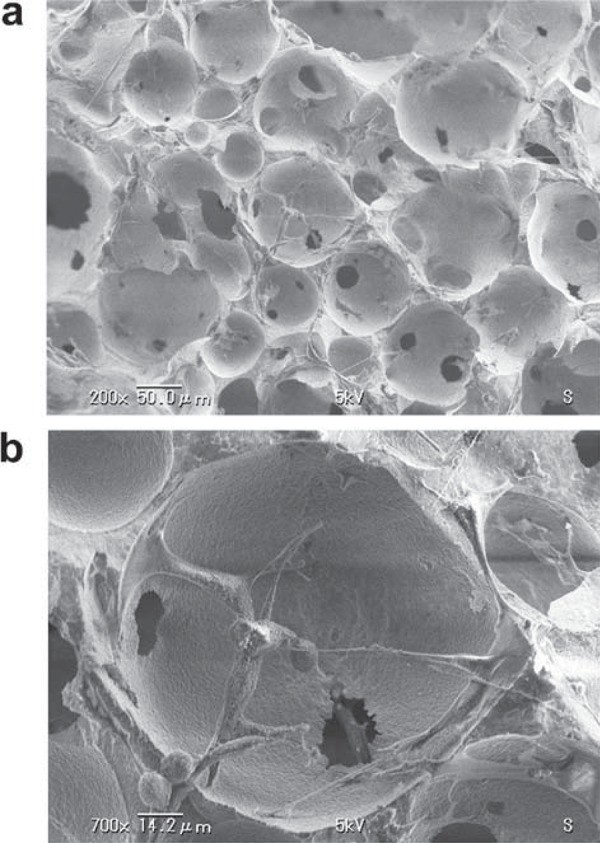
Scanning electron microscopy analysis of IP-CHA/MC3T3-E1 cells composites. a- The seeded MC3T3-E1 cells were observed in the interior walls of porous IP-CHA, 3.5 mm deep from the surface; b- Higher magnification showed that MC3T3-E1 cells attached to the porous walls

To confirm the presence of MC3T3-E1 inside IP-CHA, IP-CHA/MC3T3-E1 composites were examined by SEM after 3 days of culture. We found the seeded MC3T3-E1 cells in the interior walls of porous IP-CHA, 3.5 mm deep from the surface (Figures 3a and 3b).

### FGF-2 induced proliferation of MC3T3 cells within IP-CHA construct

**Figure 4:**
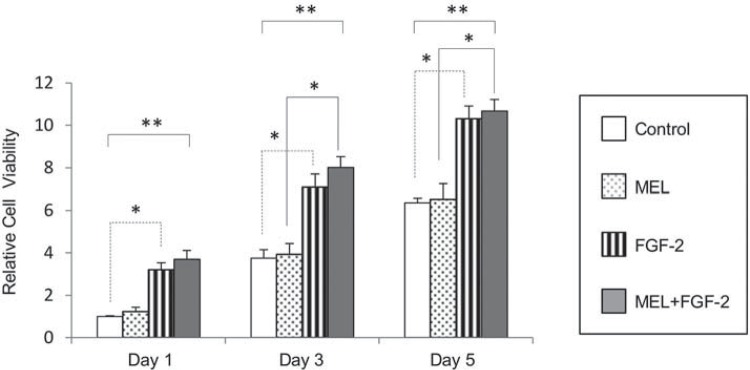
Proliferation assay of IP-CHA/MC3T3-E1 cells under the influence of FGF-2 and/or melatonin (MEL). Cell growth was significantly induced by FGF-2 treatment compared to the control and to the treatment with MEL. The combination of MEL and FGF-2 more markedly promoted the proliferation of IP-CHA/MC3T3-E1 cells. Statistical significances shown by p<0.05 and p<0.01 are indicated by * and **, respectively

To evaluate the combined effect of FGF-2 and MEL on the proliferative potential of MC3T3-E1 cells within an IP-CHA construct, IP-CHA/MC3T3-E1 composites were examined in the presence of 20 µg/ml FGF-2 and/or 200 nM MEL after 1, 3, and 5 days of culture. FGF-2 independently and significantly induced growth of MC3T3-E1 cells compared to the control on day 5 (Figure 4), whereas MEL alone showed no significant effect on cell proliferation.

### MEL main contributor to induce mRNA expression of late bone markers within IP-CHA construct

**Figure 5:**
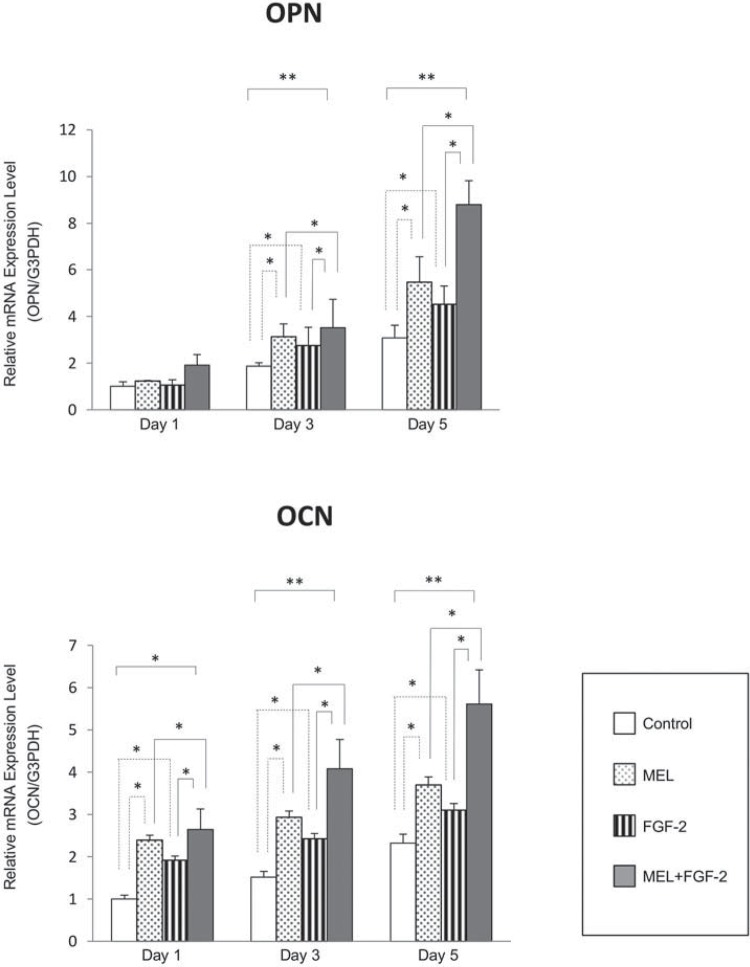
mRNA expressions of osteopontin (OPN) and osteocalcin (OCN) of IP-CHA/MC3T3-E1 cells under the influence of FGF-2 and/or melatonin (MEL). mRNA expressions of OPN and OCN were significantly enhanced by treatment with MEL from day 3 compared to the control and to the treatment with FGF-2. The effect of combined MEL and FGF-2 was significantly greater. Statistical significances shown by p<0.05 and p<0.01 are indicated by * and **, respectively

Figure 5 shows OPN and OCN expressions. Individual treatment by FGF-2 and MEL induced expression of these markers from day 5, though only MEL treatment had a significant effect (Figure 5). On the other hand, combined treatment had an even greater effect on increased OCN and OPN expression (Figure 5).

### MEL main contributor to enhance ALP activity within IP-CHA construct

**Figure 6:**
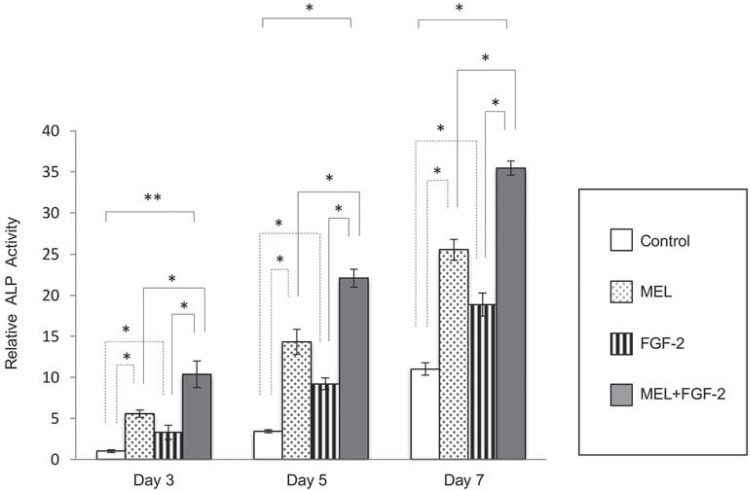
ALP enzyme activity of IP-CHA/MC3T3-E1 cells under the influence of FGF-2 and/or melatonin (MEL). ALP activity from day 3 was significantly higher in cells treated with MEL compared to the control and to those treated by FGF-2. The combination of MEL and FGF-2 markedly increased that activity. The ratio of absorbance of each sample in relation to the control sample at day 3 was observed. Statistical significances shown by p<0.05 and p<0.01 are indicated by * and **, respectively

Figure 6 shows the relative ALP enzyme activities of MC3T3-E1 cells cultured within IP-CHA constructs with MEL and/or FGF-2 on days 3, 5, and 7. ALP enzyme activity is known to be closely associated with osteoblast differentiation. Both FGF-2 and MEL independently induced ALP activity from day 3, though induction by the latter was more prominent (Figure 6). The combination resulted in significantly greater ALP enzyme activity compared to the individual treatments (Figure 6).

### Combination of FGF-2 and MEL enhanced mineralization

**Figure 7:**
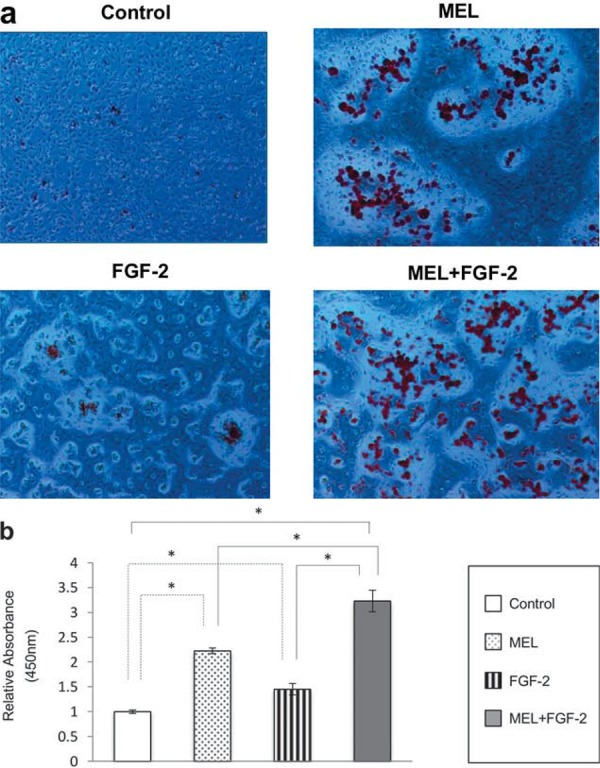
Alizarin Red Staining of MC3T3-E1 cells under the influence of FGF-2 and/or melatonin (MEL). a- Alizarin Red staining of monolayer cultures showed increased mineralization after 2 weeks of treatment with MEL or FGF-2 in osteogenic induction medium compared to the control. The combination of MEL and FGF-2 resulted in more intense staining; b- Quantified values obtained from cells cultured within IP-CHA constructs suggested a role for MEL in mineralization, while combined FGF-2 and MEL induced increased mineralization. Statistical significance shown by p<0.05 is indicated by *

Figure 7a shows Alizarin Red Staining after MC3T3-E1 cells were treated for 2 weeks with MEL and/or FGF-2 in osteogenic induction medium. MEL and FGF-2 independently stimulated extracellular calcium deposition in both monolayer cultures and treated IP-CHA/MC3T3 composites (Figures 7a and 7b). Those in combination resulted in more intense mineralization staining and higher quantified values (Figures 7a and 7b). These results indicate that the combination of FGF-2 and MEL is involved in augmentation of mineralization.

## DISCUSSION

HAp is a biocompatible material and possesses the advantage of protein adhesion whereby it can facilitate osteoblastic cell binding, proliferation, and differentiation, leading to matrix organization^12^. Fully interconnected porous HAp is thought to be a suitable candidate for transplantation of both osteoinductive agents and osteoblastic cells. In this study, we found that mouse preosteoblastic cells can penetrate and grow inside the construct. In addition, attachment of MC3T3-E1 cells to porous hydroxyapatite was well documented in a study presented by Smith, et al.^19^ (2006). These results suggest that IP-CHA blocks are suitable scaffold for osteoblastic cells.

Bone formation is a cascade of events that occur in the initial proliferation phase, followed by the mineralization phase marked by OPN and OCN expressions^14^. FGF-2 has been reported to be an eminent growth factor which is more favorable to cell proliferation than differentiation^11^, which was also shown in our study using IP-CHA constructs. The relative proliferation rate and insignificant effect of FGF-2 on the late bone markers OPN and OCN highlight its role in the proliferative phase of osteoblast activity in contrast to differentiation^11^. This phenomenon is of particular interest, because FGF-2 significantly increased the osteoblast population within the IP-CHA constructs in our study, ensuring that more cells were available for entering the maturation phase of bone formation.

MEL has been reported to positively stimulate bone formation by suppressing RANKL-mediated osteoclast formation and resultant bone resorption in the bone remodeling cycle of MC3T3-E1 cells^6^. In addition, Roth, et al.^17^ (1999) have reported that MEL can induce differentiation of MC3T3-E1 cells and mineralization of matrix in culture. These observations indicate that MEL plays a significant role in bone formation. Although MEL had no significant influence on the rate of cell growth in our study, as anticipated, its differentiation potential was largely highlighted by the mRNA expressions of the late osteogenic markers OPN and OCN. Furthermore, we found that MEL induced mineralization, as shown by Alizarin Red staining. These findings suggest that MEL is significantly involved in osteoblast differentiation and mineralization.

MEL may induce increased cellular activity and differentiation because of its activity as an inherent free radical scavenger^22^. MC3T3 cells expel various free radicals during proliferation and growth^8^, while buildup of a large amount of free radicals hampers the natural activity of MC3T3 cells, leading to inhibition of mineralization^9^. Therefore, we speculate that MEL assists MC3T3 cellular activity and mineralization by neutralizing free radicals. In the present study, the combination of FGF-2 and MEL upregulated OPN and OCN, and also increased mineralization compared to treatment with each one alone. We concluded that FGF-2 induces proliferation, which provides a larger number of cells for MEL to induce to differentiation into mature osteoblasts and therefore positively regulate mineralization.

FGF-2 was previously studied in combination with other growth factors, such as bone morphogenic proteins (BMPs), and shown to significantly stimulate cell proliferation, while BMPs alone significantly stimulated differentiation and in combination with FGF-2 increased mineralization^3^. The combination of MEL and FGF-2 in the present study may operate in a similar manner, in which FGF-2 acts as the proliferative agent and MEL as the differentiating agent. This finding may also help to consider delayed administration of MEL in a future *in vivo* model. We found that MEL induced differentiation of preosteoblasts into mature bone forming successors. These findings may help explain the findings presented by Takechi, et al.^20^ (2008), in which superior osseointegration was achieved by use of titanium screws in rat tibias after systemic administrations of MEL and FGF-2.

## CONCLUSIONS

In conclusion, FGF-2 and MEL may play a significant synergistic role to enhance mineralization of MC3T3-E1 cells within IP-CHA constructs by targeting different phases of the osteoblast lineage. Clinically, combined use of FGF-2 and MEL may be a reasonable adjunct to biomaterials for use in eminent craniofacial surgery.
